# What Predicts Mortality in Essential Tremor? A Prospective, Longitudinal Study of Elders

**DOI:** 10.3389/fneur.2018.01077

**Published:** 2018-12-07

**Authors:** Adeel Zubair, Tess E. K. Cersonsky, Sarah Kellner, Edward D. Huey, Stephanie Cosentino, Elan D. Louis

**Affiliations:** ^1^Division of Movement Disorders, Department of Neurology, Yale School of Medicine, Yale University, New Haven, CT, United States; ^2^Department of Neurology, College of Physicians and Surgeons, Columbia University, New York, NY, United States; ^3^Taub Institute for Research on Alzheimer's Disease and the Aging Brain, College of Physicians and Surgeons, Columbia University, New York, NY, United States; ^4^Department of Psychiatry, College of Physicians and Surgeons, Columbia University, New York, NY, United States; ^5^Department of Chronic Disease Epidemiology, Yale School of Public Health, Yale University, New Haven, CT, United States; ^6^Center for Neuroepidemiology and Clinical Neurological Research, Yale School of Medicine, Yale University, New Haven, CT, United States

**Keywords:** mortality, essential tremor, cognitive impairment, dementia, depression, cognitive aging

## Abstract

**Objective:** Essential tremor (ET) is among the most common neurologic diseases. Although in the past it was considered a benign condition, recent research has demonstrated increased risk of mortality. To date, however, no studies have examined predictors of mortality in ET.

**Methods:** In a longitudinal, prospective study of 141 elders with ET, we used Cox proportional-hazards models to estimate hazard ratios (HRs) for death.

**Results:** The mean baseline age was 81.1 ± 8.8 years. During the follow-up interval, 27 (19.1%) died. Average time from baseline to death was 12.3 ± 8.7 months (range = 0.3–31.2). In univariate Cox regression models, older age (HR = 1.16, *p* < 0.001), lower Montreal Cognitive Assessment score (HR = 0.88, *p* = 0.004), higher Clinical Dementia Rating (CDR) score (HR = 4.53, *p* < 0.001), higher score on the Geriatric Depression scale (GDS) (HR = 1.07, *p* = 0.048), less balance confidence (HR = 0.98, *p* = 0.006), more falls (HR = 1.11, *p* = 0.003), and more tandem mis-steps (HR = 1.53, *p* = 0.004) were associated with increased risk of mortality. In the final multivariate Cox model, older age (HR = 1.14, *p* = 0.005), higher CDR score (HR = 3.80, *p* = 0.002) and higher GDS (HR = 1.11, *p* = 0.01) were independently associated with increased risk of mortality.

**Conclusions:** This study highlights several independent predictors of mortality in elderly ET; clinicians should consider screening for depressive symptoms, assessing cognition and tracking CDR scores, and assessing balance while evaluating patients with ET.

## Introduction

Essential tremor (ET) is one of the most common neurological disorders among adults. Traditionally, ET has been thought of as a monosymptomatic disorder, characterized by kinetic arm tremor. It has an ~4% prevalence in adults older than age 40 and the prevalence is even higher among the elderly (1). Case-control studies have shown that patients with ET may develop additional motor problems (i.e., mild gait ataxia with increased risk for falls in some) and non-motor problems [i.e., mild sleep impairment, depressive symptoms, mild cognitive impairment [MCI], and dementia] (2–5). ET is a progressive disease; the tremor gradually worsens with time. This, along with the accumulation of the co-morbidities noted above, result in both a functional decline and increased frailty (6).

Studies of mortality in ET are few, with two longitudinal studies, one being retrospective and the other prospective. First, a longitudinal, retrospective study compared 266 ET patients in Rochester, MN with a historical control group; no significant increase in mortality was detected (7). However, the study had limitations, including the young age of the population (mean baseline age = 58 years with mean follow-up = 9.7 years); hence many individuals were not followed into advanced age, which is when mortality events are most likely to occur. Second, a longitudinal, prospective study of mortality compared the risk of mortality in 201 population-dwelling ET cases with 465 age-matched controls (combined mean baseline age = 73.5 ± 6.4 years); the risk of mortality was increased in ET [relative risk [RR] = 1.59, 95% confidence interval [CI] = 1.11–2.27, *p* = 0.01] (8).

Despite the observed increase in mortality (8), no studies to date have examined predictors of mortality in ET. Identification of risk factors for mortality is valuable for health care planning, and in drawing attention to features for which better treatment might have the greatest clinical consequences, including prolonged survival. We capitalized on a large, longitudinal, prospective study of elderly people with ET to examine the demographic and clinical factors associated with increased risk of mortality. *A priori*, we hypothesized that increased risk of mortality would be observed in older ET cases, male cases, those with higher baseline Clinical Dementia Rating (CDR) scores, and those with more falls and/or more gait difficulties.

## Methods

### Study Design

ET cases were enrolled in an ongoing longitudinal, prospective study of cognitive function in ET (Clinical Pathological Study of Cognitive Impairment in Essential Tremor, NINDS R01NS086736), which started enrolling on a rolling basis in July 2014. The purpose of the study was to characterize the clinical features of a cohort of ET cases using motor, neuropsychologic, and neuropsychiatric measures. Cases were recruited using advertisements on the study website and other websites (e.g., International Essential Tremor Foundation) according to the following eligibility criteria: (1) diagnosis of ET; (2) age over 55 years (although the majority >65 years); (3) no previous history of surgical interventions to treat ET; and (4) enrollment as a brain donor in the Essential Tremor Central Brain Repository and willingness to perform extensive study measures. Three assessments (baseline, 18-months, 36-months) were planned. The current analyses use data collected from the baseline and 18-months assessments, and mortality data collected through May 2017.

ET diagnoses were confirmed after enrollment based on a detailed history and videotaped neurological examination reviewed by a senior neurologist specializing in movement disorders (EDL) who applied reliable (9) and valid (10) diagnostic criteria (moderate or greater amplitude kinetic tremor during three or more videotaped activities or a head tremor in the absence of Parkinson's disease or other known causes) (11).

Each case also enrolled as a brain donor with a regularly-updated, individualized plan for postmortem brain procurement. At the time of death, cases' pre-designated next of kin contact us to arrange for brain donation. The Yale University and Columbia University Internal Review Boards approved all study procedures (#1410014757). Signed, written informed consent was obtained 184 upon enrollment in accordance with the Declaration of Helsinki.

### Study Evaluation

Trained research assistants performed all in-person study assessments in the cases' homes; in most instances, they divided the visit into two 2 h sessions over 2 days to avoid subject fatigue. The research assistants collected demographic data (e.g., age, gender, race, education) and basic clinical data (e.g., number of prescription medications, current cigarette smoker) and conducted a detailed tremor history (e.g., age of tremor onset). They also administered the Activities-specific Balance Confidence Scale [ABC-6, range of total score = 0 [most impaired]−100 [no impairment]]. The scale asks cases to rate their confidence in performing functional activities without losing balance or becoming unsteady, assessing a range of 6 situation-specific activities (e.g., reaching on tiptoes for an object, stepping on or off an escalator) (12). During the assessment, the research assistants also asked cases how many falls they had experienced during the past year. Cases also completed the Geriatric Depression Scale (GDS), a 30-item self-report measure of depressive symptoms (13). This is a self-administered measure of depressive symptoms designed for use in older persons. It focuses on the affective, rather than somatic symptoms of depression as the somatic symptoms of depression can be difficult to distinguish from symptoms of medical and neurological illness in the elderly. The subject answers yes/no questions regarding symptoms of depression. Higher scores indicate more symptoms of depression. Scores >10 reflect significant depression and scores >20 reflect severe depression.

The trained research assistants also administered a neuropsychological test battery, designed by a neuropsychologist (SC). Tests were selected for the neuropsychological test battery that required minimal motor activity so as to reduce the chance that tremor would affect test scores (14). The battery included the Montreal Cognitive Assessment (MoCA) (15), which is a brief screening test of cognition [range = 0–30 [higher reflects better cognition]] as well as tests of cognitive function within discrete cognitive domains: *memory* [California Verbal Learning Test [CLVT-II] (16), Wechsler Memory Scale Revised [WMS-R]: Logical Memory [LM] (17), Wechsler Memory Scale IV [WMS-IV]: Verbal Paired Associates [VPA] (18)], *executive function* [Wechsler Adult Intelligence Scale IV [WAIS-IV]: Digit Span Backward (19), Delis-Kaplan Executive Function System [D-KEFS]: Verbal Fluency Test [VFT], Color-Word Interference [CW], Sorting, 20-Questions [20Q] (20)], *attention* [Oral Symbol-Digit Modalities Test [SDMT] (21), WAIS-IV: Digit Span Forward (19)], *visuospatial ability* [Benton Judgment of Line Orientation [JLO] (22), Benton Facial Recognition Test [BFRT] (23), WAIS-IV: Visual Puzzles (19)], and *language* [Multilingual Aphasia Examination [MAE]: Token Test (24), Boston Naming Test [BNT] (25)]. Composite scores for each domain were calculated using the means of the following z-scores, calculated according to clinically-available normative data adjusted by age, gender, and/or education: *memory* (CVLT-II Immediate Recall and Long-Delay Free-Recall, VPA I and II, LM I and II), *executive function* [Digit Span backward, all sub-scores of each DKEFS sub-test [VFT, CW, Sorting, 20Q]], *attention* (SDMT, Digit Span Forward), *visuospatial ability* (JLO, BFRT, Visual Puzzles), and *language* (MAE Token Test, BNT).

Additionally, there was an informant interview. Informants, when available, were designated by cases, and participated in a short telephone interview during which the research assistant queried the informant on the case's level of everyday functioning using the CDR. CDR scores were rated between 0 and 3, with a score of 0 being normal, a score of 0.5 signifying MCI, and scores of 1 and higher signifying dementia (26). The CDR scores were assigned based on interview and impression of the patient in conjunction with the informant report (when available) (26). CDR scores were reviewed in diagnostic consensus conferences by a geriatric psychiatrist (EDH) and neuropsychologist (SC).

Three primary cognitive diagnoses were assigned during consensus conferences using clinical judgment and diagnostic specifications: (1) Normal Cognition; (2) MCI (CDR = 0.5 and impairment on 2 MCI-designated tests); and (3) Dementia (CDR >1 and impairment in multiple cognitive domains), as described previously (14).

Finally, the research assistants performed a videotaped neurological examination during the in-person assessment. This included one test for postural tremor and five for kinetic tremor (e.g., pouring, drinking) performed with each arm (12 tests total), the motor portion of the Unified Parkinson's Disease Rating Scale (UPDRS) (27) excluding an assessment of rigidity, and a comprehensive assessment of dystonia. EDL used the Washington Heights-Inwood Genetic Study of ET (WHIGET) tremor rating scale to rate postural and kinetic tremor during each test (0–3). These ratings resulted in a total tremor score (range = 0–36). Tremor ratings using this method are highly reliable (9) and have been validated against subject measures of tremor-related disability in ET (28), performance-based measures of functional impairment in ET (29), and objective measurements of tremor in ET (30). The videotaped assessment also included an assessment of tandem gait. Research assistants asked each case to walk tandem (place one foot in front of the other touching toe to heel) and the number of missteps during 10-steps was counted by EDL (31).

### Cause of Death

After death, data on cause of death were abstracted from death certificates. These data were supplemented by those from medical records.

### Statistical Analyses

Statistical analyses were performed using SPSS (Version 24; Chicago, IL, USA). We compared baseline demographic and clinical features in ET cases who had died vs. not died during prospective follow-up; these analyses used Student *t*-tests, chi-square tests, Fisher's exact tests and Mann-Whitney tests as appropriate. We used Cox proportional-hazards models to estimate hazard ratios (HRs) for death with 95% confidence intervals (CIs). In cases who were living, a person-years variable was calculated using the time between the baseline and the follow-up evaluation. By contrast, in those who died, person-years was the time between the baseline evaluation and the date of death. Initial models were univariate (i.e., we entered variables one at a time in a series of models) and based on these analyses, multivariate models were constructed. In these multivariate models, successive variables were added one at a time rather than *en bloc* in order to account for potential collinearity between variables (e.g., cognitive domain variables). The *p*-value for significance was set at < 0.05. We also performed a sensitivity analysis in which we excluded 48 cases with CDR >0, and using Cox proportional-hazards models examined the predictors of mortality in a cognitively normal cohort of 77 cases.

The raw data supporting the conclusions of this manuscript will be made available by the authors, without undue reservation, to any qualified researcher.

## Results

There were 141 ET cases whose baseline age was 81.1 ± 8.8 years (Table 1). During the follow-up interval, 27 (19.1%) of 141 died. Of these 27, 23 (85.2%) died after the baseline assessment and 4 (14.8%) died after the 18 months assessment. The average time from baseline assessment to most recent contact for living participants was 21.5 ± 7.8 months. The average time from baseline assessment to death was 12.3 ± 8.7 months (range = 0.3–31.2).

**Table 1:** Baseline demographic and clinical characteristics of ET cases who had died vs. did not die during prospective follow-up.

	**All ET Cases**	**Deceased**	**Living**	**Significance (Deceased vs. Living)**
	***N* = 141**	***N* = 27**	***N* = 114**
Age (years)	81.1 ± 8.8	89.2 ± 5.2	79.2 ± 8.4	***p*** **<0.001**^**a**^
Female gender	85 (60.3)	17 (63.0)	68 (59.6)	*p* = 0.76^b^
White race	139 (98.6)	27 (100.0)	112 (98.2)	*p* = 1.00^c^
Education (years)	15.5 ± 2.8	15.0 ± 3.0	15.7 ± 2.8	*p* = 0.26^a^
Number of prescription medications	6.5 ± 4.9 [5.0]	8.5 ± 5.7 [7.0]	6.0 ± 4.5 [5.0]	***p*** **=** **0.005**^**d**^
Current cigarette smoker	4 (2.8)	1 (3.7)	3 (2.6)	*p* = 0.58^c^
Age of tremor onset (years)	40.5 ± 23.0 [40.0]	46.3 ± 23.4 [55.0]	39.2 ± 22.8 [40.0]	*p* = 0.14^d^
Total tremor score	21.1 ± 6.0	21.8 ± 6.5	20.9 ± 6.0	*p* = 0.50^a^
MoCA score	23.8 ± 4.0 [25.0]	21.1 ± 4.7 [22.0]	24.3 ± 3.7 [25.0]	***p*** **<** **0.001**^**d**^
Cognitive Diagnosis^*^				***p*** **=** **0.001**^**b**^
Normal cognition	94 (67.6)	11 (40.7)	83 (74.1)
MCI	28 (20.1)	8 (29.6)	20 (17.9)
Dementia	17 (12.2)	8 (29.6)	9 (8.0)
CDR^**^^#^				***p*** **<** **0.001**^**b**^
0	77 (61.6)	6 (27.3)	71 (68.9)
0.5	34 (27.2)	9 (40.9)	25 (24.3)
1	9 (7.2)	2 (9.1)	7 (6.8)
2	5 (4.0)	5 (22.7)	0 (0.0)
3	0 (0.0)	0 (0.0)	0 (0.0)
Cognitive Domain				
Memory	−0.34 ± 1.03 [−0.14]	−1.05 ± 1.19 [−1.28]	−0.17 ± 0.91 [0.004]	***p*** **<** **0.001**^**d**^
Executive Function	−0.14 ± 0.80 [0.02]	−0.70 ± 1.00 [−0.69]	−0.01 ± 0.68 [0.10]	***p*** **=** **0.001**^d^
Attention	−0.09 ± 0.97	−0.47 ± 0.77	−0.02 ± 0.99	***p*** **=** **0.04**^**a**^
Visuospatial Abilities	0.39 ± 0.79	0.33 ± 0.75	0.41 ± 0.80	*p* = 0.64^a^
Language	−0.50 ± 0.98 [−0.32]	−0.87 ± 1.22 [−0.57]	−0.42 ± 0.90 [−0.31]	*p* = 0.12^d^
GDS	6.9 ± 5.6 [6.0]	9.0 ± 5.4 [8.0]	6.4 ± 5.6 [5.0]	***p*** **=** **0.01**^**d**^
ABC	51.5 ± 29.5 [52.5]	29.2 ± 23.0 [23.3]	56.2 ± 28.7 [61.7]	***p*** **<** **0.0001**^**d**^
Number of falls reported during past year	1.3 ± 3.1	2.9 ± 6.3 [2.0]	1.3 ± 2.7 [0.0]	*p* = 0.056^d^
Number of tandem missteps	5.7 ± 4.1 [6.0]	9.6 ± 1.4 [10]	4.9 ± 4.0 [4.0]	***p*** **<** **0.0001**^**d**^

*Values are number (percentage) or mean ± standard deviation [median]*.

* *2 individuals had cognitive impairment but were not classified as MCI or dementia*.

** *Some values are missing if no informant was available*.

# *Percentage represents a column percentage*.

*Bolded values are statistically significant (p < 0.05)*.

*ABC, Activities-specific Balance Confidence Scale Short Form; CDR, Clinical Dementia Rating; ET, essential tremor; GDS, Geriatric Depression Scale; MCI, mild cognitive impairment; MoCA, Montreal Cognitive Assessment*.

a *Student t-test*.

b *Chi-square test*.

c *Fisher's Exact test*.

d *Mann-Whitney test*.

The 27 ET cases who died differed with respect to numerous baseline demographic and clinical characteristics from the 114 ET cases who did not die during follow-up (Table 1). Those who died had an older baseline age, had been on more prescription medications, had more cognitive impairment (i.e., lower MoCA score, larger proportion who were demented, higher CDR score, and lower scores in the cognitive domains of memory, executive function and attention), had more depressive symptoms (i.e., higher GDS score), and had more gait and balance difficulty (i.e., lower ABC score, marginally more falls during the past year, more tandem missteps, Table 1). The two groups did not differ significantly with respect to gender, education, cigarette smoking, age of tremor onset or total tremor score (Table 1).

**Figure 1:**
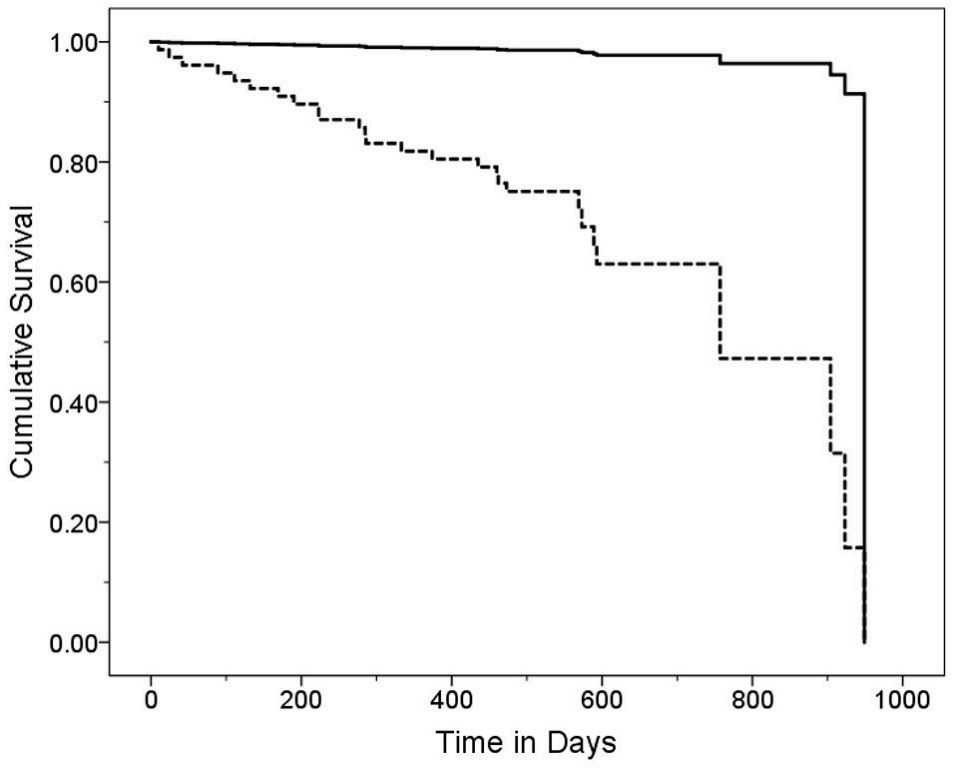
Survival Curve by Age Category. ET cases were stratified into two age groups based on the median age (80 years). Older ET cases (solid line) had a mean ± standard deviation [median] age = 87.9 ± 4.3 [88.0] years and younger ET cases (dashed line) had a mean ± standard deviation [median] age = 73.6 ± 5.9 [75.0] years. Hazards ratio (95% Confidence Interval) in Cox proportional-hazards model = 20.43 (2.75–151.95), *p* = 0.003.

**Figure 2:**
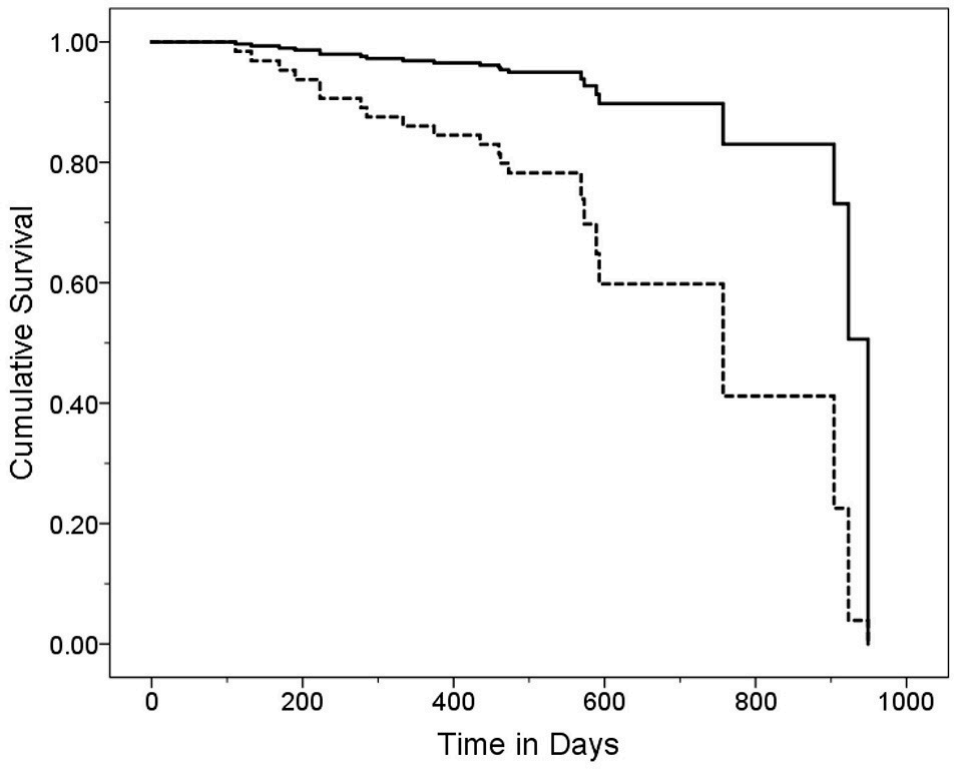
Survival Curve by CDR Category. ET cases were stratified into two CDR score groups. Cases with higher CDR scores (solid line) had a mean ± standard deviation [median] CDR score = 0.75 ± 4.7 [0.5] years and cases with lower CDR scores (dashed line) had a mean ± standard deviation [median] CDR score = 0.0 ± 0.0 [0.0] years. Hazards ratio (95% Confidence Interval) in Cox proportional-hazards model = 4.76 (1.73–13.12), *p* = 0.003.

**Figure 3:**
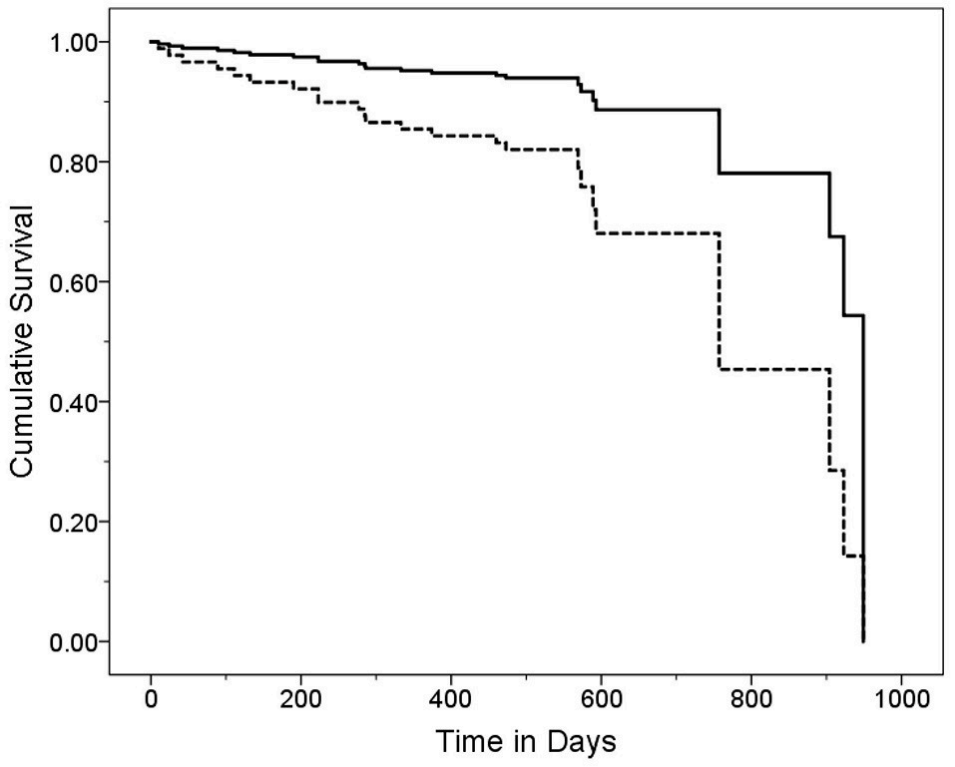
Survival Curve by GDS Category. ET cases were stratified into two GDS categories based on the median GDS score (GDS = 5). Cases with higher GDS scores (solid line) had a mean ± standard deviation [median] GDS score = 10.9 ± 5.0 [9.0] years and cases with lower CDR scores (dashed line) had a mean ± standard deviation [median] GDS score = 2.6 ± 1.7 [3.0] years. Hazards ratio (95% Confidence Interval) in Cox proportional-hazards model = 3.20 (1.18–8.67), *p* = 0.02.

In a series of univariate Cox regression models, we entered variables one at a time. These analyses revealed that older age (HR = 1.16, *p* < 0.001), lower MoCA score (HR = 0.88, *p* = 0.004), worse cognitive diagnosis (normal, MCI, dementia, HR = 2.20, *p* = 0.001), higher CDR score (HR = 4.53, *p* < 0.001), more difficulty with memory (HR = 0.52, *p* = 0.001), more difficulty with executive function (HR = 0.39, *p* < 0.001), more difficulty with language (HR = 0.51, *p* = 0.005), more depressive symptoms (HR = 1.07, *p* = 0.048), and more balance issues [lower ABC score [HR = 0.98, *p* = 0.006], more falls during past year [HR = 1.11, *p* = 0.003] and more tandem mis-steps [HR = 1.53, *p* = 0.004])] were associated with increased risk of mortality (Table 2). In our final multivariate Cox model, older age (HR = 1.14, *p* = 0.005), higher CDR score (HR = 3.80, *p* = 0.002), and higher GDS score (HR = 1.11, *p* = 0.01) were each independently associated with increased risk of mortality (Table 3). Survival curves are shown for age (Figure 1), CDR score (Figure 2), and GDS score (Figure 3).

**Table 2:** Predictors of mortality in ET cases (Univariate Cox proportional-hazards models).

**Variable**	**Beta**	**HR**	**95% CI**	**Significance**
Age (years)	0.15	1.16	1.08–1.24	***p*** **<0.001**
Female gender	0.12	1.13	0.51–2.51	*p* = 0.76
White race	3.03	20.70	0.00–19162879.05	*p* = 0.66
Education (years)	−0.05	0.95	0.82–1.11	*p* = 0.53
Number of prescription medications	0.011	1.01	0.95–1.08	*p* = 0.76
Current cigarette smoker	0.47	1.59	0.24–11.87	*p* = 0.65
Age of tremor onset (years)	0.009	1.01	0.99–1.03	*p* = 0.34
Total tremor score	0.01	1.01	0.95–1.09	*p* = 0.70
MoCA score	−0.13	0.88	0.80–0.96	***p*** **=** **0.004**
Cognitive Diagnosis (Normal cognition, MCI, Dementia)	0.79	2.20	1.38–3.52	***p*** **=** **0.001**
CDR	1.51	4.53	2.48–8.28	***p*** **<0.001**
Cognitive Domain				
Memory	−0.65	0.52	0.36–0.76	***p*** **=** **0.001**
Executive Function	−0.94	0.39	0.24–0.63	***p*** **<** **0.001**
Attention	−0.43	0.65	0.37–1.13	*p* = 0.12
Visuospatial Ability	−0.23	0.80	0.47–1.36	*p* = 0.40
Language	−0.67	0.51	0.32–0.82	***p*** **=** **0.005**
GDS	0.06	1.07	1.001–1.14	***p*** **=** **0.048**
ABC	−0.02	0.98	0.96–0.99	***p*** **=** **0.006**
Number of falls reported during past year	0.11	1.11	1.04–1.20	***p*** **=** **0.003**
Number of tandem mis-steps	0.43	1.53	1.14–2.06	***p*** **=** **0.004**

*Bolded values are statistically significant (p < 0.05)*.

*ABC, Activities-specific Balance Confidence Scale Short Form; CDR, Clinical Dementia Rating; CI, confidence interval; ET, essential tremor; GDS, Geriatric Depression Scale; HR, Hazards ratio; MCI, mild cognitive impairment; MoCA, Montreal Cognitive Assessment*.

**Table 3:** Predictors of mortality in ET cases (multivariate Cox proportional-hazards model).

	**Beta**	**HR**	**95% CI**	**Significance**
Age (years)	0.13	1.14	1.04–1.25	***p*** **=** **0.005**
CDR	1.34	3.80	1.64–8.83	***p*** **=** **0.002**
GDS	0.10	1.11	1.02–1.20	***p*** **=** **0.01**

*Bolded values are statistically significant (p < 0.05)*.

*CDR, Clinical Dementia Rating; CI, confidence interval; GDS, Geriatric Depression Scale; HR, Hazards ratio*.

We also performed a sensitivity analysis in which we only included 77 ET cases with CDR = 0, and using Cox proportional-hazards models, examined the predictors of mortality in a cognitively normal cohort of cases. In univariate models, the strongest predictors of mortality were older age (HR = 1.13, *p* = 0.066) and more tandem mis-steps (HR = 1.28, *p* = 0.096). The reduced sample size limited our ability to draw conclusions from multivariate modeling.

Cause of death was known in 19 (73.1%) of 26 cases: cardiac/pulmonary (*n* = 9), metastatic cancer (*n* = 3), subdural hematoma in the setting of a fall (*n* = 2), other causes (e.g., sepsis, pneumonia, end stage renal disease, stroke, natural causes, *n* = 5).

## Discussion

In this large, longitudinal, prospective study of 141 elders with ET, we examined the baseline factors that predicted risk of mortality. *A priori*, we hypothesized that increased risk of mortality would be observed in ET cases who were older at baseline, male, had higher baseline CDR score, and had increased falls and/or increased gait difficulties at baseline. There were 27 deaths in our cohort during the follow-up interval.

In our final multivariate model, we determined that older baseline age, higher baseline CDR score and higher baseline GDS were independent predictors of mortality in patients with ET. In a secondary analysis of cognitively normal cases, more tandem mis-steps was marginally predictive of increased risk of mortality.

The association with age is not unexpected; each year was associated with a 14% increased risk of mortality. Multiple studies have shown that age is an independent predictor for mortality in a plethora of different diseases (32, 33).

Higher CDR scores were also associated with increased risk of mortality in patients with ET. Indeed, with every incremental increase in the CDR, risk of mortality increased 3.80-times. An increased CDR score signifies more functional cognitive impairment, and higher CDR scores likely reflect the presence of a comorbid and fatal neurodegenerative disease such as Alzheimer's disease (26, 34). Cognitive dysfunction may influence gait, and poor judgment can lead to patients placing themselves into situations that increase their risk of injuring themselves. Patients with cognitive impairment are also not able to properly use assistive devices which can also lead to situations which can increase mortality.

We also found that higher GDS was a predictor of increased risk of mortality in ET, as has been demonstrated in other cohorts of elderly patients with various comorbidities (35, 36). Studies have demonstrated an association between ET and depressive symptoms (37). The GDS screens for depressive symptoms; patients with depressive symptoms can have multiple risk factors for increased mortality including decreased appetite, lack of motivation, non-compliance with medical therapy, and social withdrawal. Worsened health and greater medical co-morbidity are also associated with the development of depressive symptoms (38). Screening for depression is important in ET. We have previously shown that greater depressive symptoms in ET are associated with lower medication adherence (39), an amplification of tremor-related embarrassment (40), reduced quality of life (41), and lower receptivity to certain therapeutic interventions (e.g., deep brain stimulation surgery) (42). Now we show that depressive symptoms are associated with increased risk of mortality. Depression is treatable. Hence, these data further indicate the importance of evaluating depression in patients with ET.

One caveat is that CDR is a powerful predictor of mortality, and it likely captures cases who have a concomitant neurodegenerative condition. However, among those with CDR score of 0, other aspects of the disease itself such as balance issues are likely to retain predictive utility. Indeed, we performed a sensitivity analysis in which we excluded cases with CDR >0, and using Cox proportional-hazards models, examined the predictors of mortality in a cognitively normal cohort of 77 cases. In univariate models, the strongest predictors of mortality were older age (HR = 1.13, *p* = 0.066) and more tandem mis-steps (HR = 1.28, *p* = 0.096). Of interest is that the cause of death in 2 of 19 cases was subdural hematoma in the setting of a fall.

When we revisit our hypotheses, male gender was not associated with increased risk of mortality in the current sample. Also, although in univariate analyses increased falls and/or increased gait difficulties were associated with risk of mortality, in the final model, these were not independent predictors. During multivariate Cox modeling, we found that older age, more depressive symptoms and more reported falls were independent predictors of mortality, but when we included CDR score in the same model, the number of falls fell out. Similarly, multivariate Cox modeling, we found that older age, more depressive symptoms, and more tandem mis-steps were independent predictors of mortality, but when we included CDR score in the model, the number of falls fell out. In sensitivity analyses of cognitively normal cases, however, tandem mis-steps was among the strongest predictors of mortality.

In contrast to the situation for ET, in which there have been no studies of the predictors of mortality, there are numerous such studies for Parkinson's disease (43–46). One question is how the predictors identified here for ET differ from those identified in studies of Parkinson's disease. In the Parkinson's disease studies as well, older age (43, 45, 46), cognitive impairment (45, 46), and gait difficulty (46) have been identified as predictors of mortality. This serves to reinforce that these features are of predictive importance across neurological conditions of late life. Older age of onset/diagnosis, identified in several studies of Parkinson's disease (45, 46), was not an independent predictor in ET. While psychotic symptoms have also been identified as predictive of mortality in Parkinson's disease (45, 46), we are unaware of an association between depressive symptoms in Parkinson's disease and risk of mortality, as we identified here for ET.

These findings should be interpreted in the context of several limitations. First, the duration of follow-up is somewhat limited. With longer follow-up and a larger number of deaths, more subtle associations might be detectable. Despite this, numerous significant associations were detected. Second, the sample size was limited (*n* = 141); despite this, as noted above, numerous significant associations were detected in our univariate models (Table 2). Third, the cases self-referred to our research study and signed up to be brain donors; hence, they may not be broadly representative of ET. Fourth, our measure of medical co-morbidity was the number of prescription medications; future studies should include more robust measures of co-morbidity. Fifth, although our study examined tremor severity as a predictor of mortality, our measure of severity, the total tremor score, was based on an ordinal clinical rating scale. Quantitative computerized tremor analysis and other quantitative assessments provide a more precise metric of tremor severity and future studies should consider incorporating such a measure. Sixth, the current analyses were focused on a single outcome, mortality. As the study is a longitudinal, prospective one, future analyses could focus on the predictors of additional outcomes (e.g., incident dementia); these analyses will require a longer follow-up interval to generate a reasonable number of outcome events. Finally, we did not enroll a control group; however, we have previously performed a prospective, longitudinal study in which we compared risk of mortality in ET cases to that of controls (8). The purpose of the current analyses was to assess predictors of increased mortality among ET cases. In this sense, the study is similar to numerous studies of Parkinson's disease from around the world that aimed to assess predictors of mortality, in which cases but no controls were enrolled (43–47).

This study also had numerous strengths including (1) the longitudinal, prospective design, (2) the careful assignment of ET diagnoses and exclusion of other diagnoses by a senior movement disorders neurologist, (3) the detailed cognitive assessment with careful assignment of CDR scores and cognitive diagnoses by a neuropsychologist and geriatric psychiatrist, (4) and the inclusion of numerous baseline demographic and clinical variables as possible predictors. It is also the only study to our knowledge to examine the predictors of mortality among patients with ET and one of only two longitudinal prospective studies of mortality in ET (8).

The clinical implications of this study are that clinicians can identify ET patients who are at increased risk for mortality. While age and CDR are not modifiable risk factors, depression is. Identification and treatment of patients with depressive symptoms is important and can lead to improved outcomes for patients. Among cognitively-normal ET cases, difficulty with balance (i.e., more tandem mis-steps) may be predictive of morality.

ET has traditionally been viewed as a benign disease; more recent literature is changing this view (8, 48). Recent prospective data suggest an increased risk of mortality in patients with ET (8). This study serves to highlight several independent predictors of mortality in elderly ET patients. Clinicians should consider screening for depressive symptoms, assessing cognition and tracking CDR scores, and assessing balance while evaluating patients with ET.

## Author Contributions

AZ, EL, SC, and EH contributed to the design of the research. TC and SK contributed to the implementation of the research. AZ, TC, SK, and EL contributed to the analysis of the research. AZ, TC, SK, SC, EH, and EL contributed to the writing of the manuscript.

### Conflict of Interest Statement

The authors declare that the research was conducted in the absence of any commercial or financial relationships that could be construed as a potential conflict of interest.
